# Isolation and Antitrypanosomal Characterization of Furoquinoline and Oxylipin from *Zanthoxylum zanthoxyloides*

**DOI:** 10.3390/biom10121670

**Published:** 2020-12-13

**Authors:** Aboagye Kwarteng Dofuor, Frederick Ayertey, Peter Bolah, Georgina Isabella Djameh, Kwaku Kyeremeh, Mitsuko Ohashi, Laud Kenneth Okine, Theresa Manful Gwira

**Affiliations:** 1 West African Center for Cell Biology of Infectious Pathogens, University of Ghana, P.O. Box LG54, Legon, Accra, Ghana; akdofuor@st.ug.edu.gh (A.K.D.); kennieo1951@yahoo.com (L.K.O.); 2 Department of Biochemistry, Cell and Molecular Biology, University of Ghana, P.O. Box LG54, Legon, Accra, Ghana; 3 Centre for Plant Medicine Research, P.O. Box 73, Mampong-Akuapem, Ghana; fredayertey@gmail.com (F.A.); peter.bolah@uds.edu.gh (P.B.); 4 Department of Parasitology, Noguchi Memorial Institute for Medical Research, University of Ghana, P.O. Box LG 581, Legon, Accra, Ghana; ginabeldjameh@yahoo.com (G.I.D.); mitsukoohashi0605@gmail.com (M.O.); 5 Department of Chemistry, University of Ghana, P.O. Box LG 56, Legon, Accra, Ghana; kkyeremeh@ug.edu.gh; 6 Department of Environmental Parasitology, Tokyo Medical and Dental University, Tokyo 113-8510, Japan

**Keywords:** skimmianine, 9-oxo-ODA, cell cycle, *Trypanosoma brucei*, *Z. zanthoxyloides*, oxidative stress

## Abstract

In the absence of vaccines, there is a need for alternative sources of effective chemotherapy for African trypanosomiasis (AT). The increasing rate of resistance and toxicity of commercially available antitrypanosomal drugs also necessitates an investigation into the mode of action of new antitrypanosomals for AT. In this study, furoquinoline 4, 7, 8-trimethoxyfuro (2, 3-b) quinoline (compound **1**) and oxylipin 9-oxo-10, 12-octadecadienoic acid (compound **2**) were isolated from the plant species *Zanthoxylum zanthoxyloides* (Lam) Zepern and Timler (root), and their in vitro efficacy and mechanisms of action investigated in *Trypanosoma brucei* (*T. brucei*), the species responsible for AT. Both compounds resulted in a selectively significant growth inhibition of *T. brucei* (compound **1**, half-maximal effective concentration EC_50_ = 1.7 μM, selectivity indices SI = 74.9; compound **2**, EC_50_ = 1.2 μM, SI = 107.3). With regards to effect on the cell cycle phases of *T. brucei*, only compound **1** significantly arrested the second growth-mitotic (G2-M) phase progression even though G2-M and DNA replication (S) phase arrest resulted in the overall reduction of *T. brucei* cells in G0-G1 for both compounds. Moreover, both compounds resulted in the aggregation and distortion of the elongated slender morphology of *T. brucei*. Analysis of antioxidant potential revealed that at their minimum and maximum concentrations, the compounds exhibited significant oxidative activities in *T. brucei* (compound **1**, 22.7 μM Trolox equivalent (TE), 221.2 μM TE; compound **2**, 15.0 μM TE, 297.7 μM TE). Analysis of growth kinetics also showed that compound **1** exhibited a relatively consistent growth inhibition of *T. brucei* at different concentrations as compared to compound **2**. The results suggest that compounds **1** and **2** are promising antitrypanosomals with the potential for further development into novel AT chemotherapy.

## 1. Introduction

African trypanosomiasis (AT), a tsetse-transmitted disease of humans and livestock caused by the protozoan parasites of the genus *Trypanosoma*, is of serious health and economic concerns to humans and livestock of various sub-Saharan African countries [1,2]. In the absence of vaccines, chemotherapy remains the only practical means to control African trypanosomes [3]. However, drug resistance, side effects, and difficulty in regimen application pose serious challenges to chemotherapy [4,5,6,7]. To meet the need for novel, less toxic and more efficient chemotherapy in African trypanosomiasis, the urgency for alternative sources of potential antitrypanosomals remains considerably high. In this regard, plant-based natural products may hold significant potential as alternative chemotherapeutic agents for the control of AT [8,9,10,11].

The Rutaceae family of citrus flowering plants that includes *Teclea*, *Ruta* and *Zanthoxylum* genera are of significant medicinal and pharmacological properties. Furoquinoline alkaloids are a group of phytochemicals produced in abundance by members of the Rutaceae family. Furoquinolines from *Teclea afzelii* Engl. and *Ruta chalepensis* L have reported antiplasmodial and antifungal properties [12,13]. The antitrypanosomal activities of *Zanthoxylum zanthoxyloides* (Lam.) Zepern. and Timler (*Z. zanthoxyloides*) in *Trypanosoma brucei* (*T. brucei*) have also been reported [14]. Even though these studies suggest that furoquinoline alkaloids may play critical roles in the antimicrobial properties of Rutaceae, furoquinolines responsible for the potential antitrypanosomal activities of Rutaceae have not been identified.

The alkaloid 4,7,8-trimethoxyfuro(2,3-b) quinoline, commonly referred to as skimmianine, is probably the best known and one of the most widely distributed furoquinolines previously isolated and characterized in *Esenbeckia leiocarpa* Engl [15] and *Zanthoxylum nitidum* (Roxb) DC [16]. The biosynthesis of this compound may proceed through a series of cyclization, alkylation, oxidative cleavage, and hydroxylation reactions from the simplest furoquinoline alkaloid dictamnine [17]. It has been reported to possess potent antiplasmodial and anti-acetylcholinesterase activities [16,18]. Despite these promising pharmacological properties, its potential contribution towards the antitrypanosomal properties of the Rutaceae family has not been explored.

Plant oxylipins are a broad group of phytochemicals whose synthesis originate from the oxygenation of polyunsaturated fatty acids. The family consists of several hydroperoxides, volatile aldehydes, divinyl ethers, hydroxy-acids, oxo-acids, keto-acids, and related plant hormones, most of which are involved in physiological processes such as the regulation of stress and defense mechanisms [19]. Analysis of the transcriptome of *Zanthoxylum dissitum* Hemsl and *Zanthoxylum armatum* var. *novemfolius* identified a broad range of oxylipin genes involved in seed germination and flower development [20,21]. However, direct isolation and identification of oxylipin from *Zanthoxylum* has not yet been reported.

As a major plant oxo-acid oxylipin, 9-oxo-ODA is derived from conjugated linoleic acid through the action of lipoxygenases. It is an abundant phytochemical in tomatoes, from which it was extracted and shown to be an anti-dyslipidemic agent involved in the activation of peroxisome proliferator-activated receptor alpha (PPARα) target genes in mouse hepatocytes [22]. This PPARα-mediated function may have a metabolic significance in trypanosomes since PPARs are key regulators of lipid metabolism in *T. cruzi* [23]. However, a reported activity of oxylipins in any species of trypanosomes is currently non-existent.

Obtaining insights into the mechanisms of action of antitrypanosomals remains a challenge despite considerable progress with commercially available human African antitrypanosomal drugs [24,25]. In the present study, skimmianine and 9-oxo-ODA were isolated from the root of *Z. zanthoxyloides*, one of the most important plant species of *Zanthoxylum* on the African continent. Both compounds were shown to exhibit potent antitrypanosomal efficacies worthy of consideration for further investigation and development. The results further corroborate *Z. zanthoxyloides* as a key species of the Rutaceae family with promising chemotherapeutic potential against African trypanosomes.

## 2. Materials and Methods

### 2.1. Culture of Parasites and Mammalian Cell Lines

Blood stream forms of the subspecies *T. brucei brucei* (*T. b. brucei*) (GUTat 3.1 strains) were cultured in vitro to the logarithm phase using Hirumi’s Modified Iscove’s Medium (HMI9, Thermo Fisher Scientific, Oxford, UK) with 10% fetal bovine serum (Thermo Fisher Scientific) at 5% CO_2_ and 37 °C. Mouse macrophages (RAW 264.7 cell line) were cultivated in vitro to the logarithm phase using Dulbecco‘s Modified Eagle Media (DMEM, Thermo Fisher Scientific) with 10% fetal bovine serum at 5% CO_2_ and 37 °C.

### 2.2. Crude Extraction and Fractionation of Plants

The root of *Z. zanthoxyloides* was collected from the arboretum of the Center for Plant Medicine Research (CPMR), Mampong-Akuapem, Ghana. It was authenticated and given the voucher specimen number CPMR 4120/4121/4122. Crude extracts and fractions were prepared from the air-dried pulverized plant material (1 kg), according to a modified version of the Kupchan method of solvent extraction as described in detail in our previous studies [14]. Briefly, total crude extract (4 g) was prepared by cold maceration of the air-dried pulverized plant material in absolute methanol and dichloromethane from which other solvent fractions (hexane, 0.6 g; dichloromethane, 0.3 g; methanol, 0.25 g; water-butanol, 1.5 g) were obtained. The method has been comprehensively illustrated in Supplementary Figure S2.

### 2.3. Chromatographic and Spectrometric Analysis

Chromatographic, spectrometric, and spectroscopic analyses were performed by following the procedures and conditions employed in our previous studies [14,26]. Analytical normal phase silica-coated thin layer chromatography (TLC) was run on both Kupchan and gravity column fractions to estimate the levels of polarity and purity of all fractions, and to determine the appropriate solvent systems for silica gel gravity column chromatography. The solvent systems used in TLCs were selected based on the specific Kupchan fractions under investigation. For the dichloromethane fraction, the solvents used were ethyl acetate, dichloromethane, hexane, and occasionally methanol to facilitate the movement of highly polar compounds on normal phase. Apart from observing TLC plates under both long (365 nm) and short (254 nm) UV wavelengths, phytochemical screening of TLC spots was conducted using a number of reagents, such as iodine, ninhydrin, Dragendorff, and antimony (III) chloride. For the ninhydrin and antimony (III) chloride tests, TLC spots were developed in 10% H_2_SO_4_ with heating at 110 °C. The data obtained from TLC runs were used to set up gravity column chromatography. Appropriate solvent systems for silica gel gravity column chromatography were selected following successful TLC runs for further purification of Kupchan fractions. The column used for the silica gel gravity chromatographic step was 120 cm long and 2.5 cm wide with ethyl acetate, hexane, and methanol routinely used as mobile phases. Briefly, the 0.3 g dichloromethane fraction (FD) fraction obtained from the *Z. zanthoxyloides* Kupchan solvent partitioning (Figure S2) was loaded on a glass column containing silica gel packed to the 80 cm mark. The column was subjected to gradient elution using a mixture of *n*-hexane and ethyl acetate (90/10, 80/20, 70/30, 60/40, 50/50, 40/60, 30/70, 20/80, and 0/100). The remaining compounds on the silica column were then flushed out using 100% methanol. Semi-preparative HPLC purifications were carried out using a Phenomenex Luna reverse-phase (C18 250 × 10 mm, L × i.d.) column connected to a Waters 1525 Binary HPLC pump chromatograph with a 2998 photodiode array detector (PDA), column heater, and in-line degasser. Detection was achieved on-line through a scan of wavelengths from 200 to 400 nm, using a solvent system of A = 80/20 (H_2_O/CH_3_CN) and B = 95/5 (CH_3_CN/H_2_O) in 30 min and held in 100% CH_3_CN for 20 min (Figure S1). About 4.6 and 3.8 mg, respectively, of compounds **1** and **2** were obtained after 48 h injections. Gas chromatography-mass spectrometric (GC-MS) analysis was performed using a PerkinElmer GC Clarus 580 Gas Chromatograph (PerkinElmer, Waltham, MA, USA) interfaced to a Mass Spectrometer PerkinElmer (Clarus SQ 8S) equipped with Elite-5MS (5% diphenyl/95% dimethyl polysiloxane) fused to a capillary column (L × I.D. 30 m × 0.25 mm, df 0.25 μm). The oven temperature was programmed from 40 °C with an increase of 3 °C/min to 90 °C, then 10 °C/min to 240 °C and holding for 15 min at 240 °C. For GC-MS detection, an electron ionization system was operated in electron impact mode with ionization energy of 70 eV. Helium gas (99.999%) was used as a carrier gas at a constant flow rate of 1 mL/min, and an injection volume of 1 μL was employed. The injector temperature was maintained at 250 °C with an ion-source temperature of 150 °C. Mass spectra were taken at 70 eV with a scan interval of 0.1 s and fragments from 45 to 450 Da. The solvent delay was 0 to 2 min, and the total GC-MS running time was 46.67 min. The mass-detector used in this analysis was TurboMass (PerkinElmer, Waltham, MA, USA) and the software adopted to handle mass spectra and chromatograms was a TurboMass ver-6.1.0. Interpretation on mass-spectrum was conducted using the database of National Institute of Standard and Technology (NIST) having more than 62,000 patterns. Compounds **1** and **2** were identified as 4,7,8-trimethoxyfuro (2,3-b) quinoline and 9-oxo-10,12-octadecadienoic acid (9-oxo-ODA) respectively. Mid-infrared (IR) spectroscopy was performed with the Universal Attenuated Total Reflectance (UATR) spectrometer using the following PerkinElmer specifications: spectrum version = 10.03.09; model = spectrum 2; serial number = 94,133; number of scans = 24; resolution = 4. All solvents were HPLC grade.

### 2.4. Analysis of Cell Viability and Cytotoxicity

*T. b. brucei* were seeded at a density of 3 × 10^5^ cells/mL in 96-well plates in a two-fold dilution of compounds and incubated for 24 h. Normal mouse macrophages (RAW 264.7) were plated at a density of 3 × 10^5^ cells/mL for 48 h to allow for adherence to plates before compounds were added to cells in a two-fold dilution and incubated for another 24 h. The alamarBlue dye (10% *v*/*v*) was added to wells and incubated for another 24 h. Experiments were run in triplicates. Spectrophotometric absorbance was recorded at a wavelength of 570 nm. Diminazene aceturate (Sigma-Aldrich, Kent, UK) a known antitrypanosomal drug, was used as a positive control.

### 2.5. Analysis of Cell Cycle

Cell cycle analysis of *T. b. brucei* was performed by relying on the DNA-binding properties of propidium iodide as previously described [14]. Cells were seeded at a density of 3.0 × 10^5^ cells/mL at the half-maximal effective concentration (EC_50_) values of compounds for 24 h. Ethanol (final concentration of 70%) was used as a fixative for the cells. Phosphate-buffered saline (PBS) and guava cell cycle reagent (containing propidium iodide) were used for the washing and staining of parasites, respectively. Distribution of cells at distinct cell cycle phases was measured with the BD LSRFortessa X-20 flow cytometer (BD Biosciences, San Jose, CA, USA).

### 2.6. Fluorescence Microscopy

Effect of compounds on parasite morphology was investigated microscopically by following the same conditions and protocols described in our previous study [26]. *T. b. brucei* cells were treated with compounds at the EC_50_ values for 24 h. Paraformaldehyde (final concentration of 4%) was used as the fixative and DAPI was used for the visualization of the nucleus and kinetoplast. Cells were washed regularly and alternately as required using PBS and PBST (PBS with 0.1% Triton). Cells were mounted with 90% glycerol in PBS for observation with the Olympus DP72 reflected fluorescence microscope. Data were analyzed with the cellSens standard imaging software and Adobe Photoshop CS6 (Adobe Inc., San Jose, CA, USA). 

### 2.7. Antioxidant Potential Analysis

The ABTS (2,2′-azino-bis (3-ethylbenzthiazoline-6-sulfonic acid) antioxidant assay kit (Sigma-Aldrich, UK) was used for the investigation of antioxidant capacity of compounds by following the manufacturer’s protocols with slight modifications. The assay involves the formation of ferryl myoglobin radical from metmyoglobin and hydrogen peroxide, which then oxidizes ABTS to a soluble radical cation that absorbs optimally at 405 nm. Antioxidants generally inhibit the production of the ABTS radical in a dose-dependent manner, thereby proportionally decreasing the absorbance at 405 nm. However, oxidants would result in a dose-dependent increase in absorbance at 405 nm due to an increased production of the ABTS radical and other potential oxidant-mediated intracellular reactive oxygen species (ROS) released within the parasites, thereby significantly facilitating oxidation within *T. brucei*. Briefly, *T. b. brucei* cells were seeded at a density of 3 × 10^5^ cells/mL on 96-well plates in a two-fold dilution of compounds. Myoglobin was added to each well and incubated for 24 h. ABTS was added to each well and incubated for approximately 5 min at room temperature. After inactivating the reaction by adding a stop solution, absorbance was read at 405 nm. Trolox ((±)-6-hydroxy-2,5,7,8-tetramethylchromane-2-carboxylic acid) was used as the positive control antioxidant. Experiments were performed in duplicates. Oxidative concentrations were calculated from the linear regression of the Trolox standard curve.

### 2.8. Antitrypanosomal Sensitivity Analysis

*T. b. brucei* cells were grown to a density of 2 × 10^6^ cells/mL and split into fresh media with the antitrypanosomal compounds at 1.5 × 10^5^ cells/mL. Cells were monitored and counted for 5 days, splitting into fresh media with the compounds at 1.5 × 10^5^ cells/mL as and when was required by the growth of cells using a maximum threshold of 5 × 10^5^ cells/mL. The addition of compounds at different doses to cells was regulated by the viability or number of parasites at any point in time: a minimum threshold of 1 × 10^5^ cells/mL was set to regulate the exposure of parasites to compounds.

### 2.9. Statistical Analysis

Data from cell viability and antioxidant activity assays were analyzed with GraphPad Prism version 5. The half-maximal effective concentration (EC_50_) was calculated as the concentration that caused a 50% reduction in cell viability. EC_50_ values were calculated from a non-linear regression model using the Hill function. Bar charts for cell cycle were generated through analysis with BD FACSDiva 8.0.1 (BD Biosciences, San Jose, CA, USA) and FlowJo V10 (FlowJo LLC, Ashland, OR, USA) analysis of cell cycle percentage counts was carried out with GraphPad Prism version 5 (GraphPad Software Inc., San Diego, CA, USA) using the unpaired *t*-test. *p*-values < 0.05 were considered to be significant.

## 3. Results

### 3.1. Bioactivity-Guided Chromatography, Spectrometry, and Spectroscopy of Compounds

**Figure 1:**
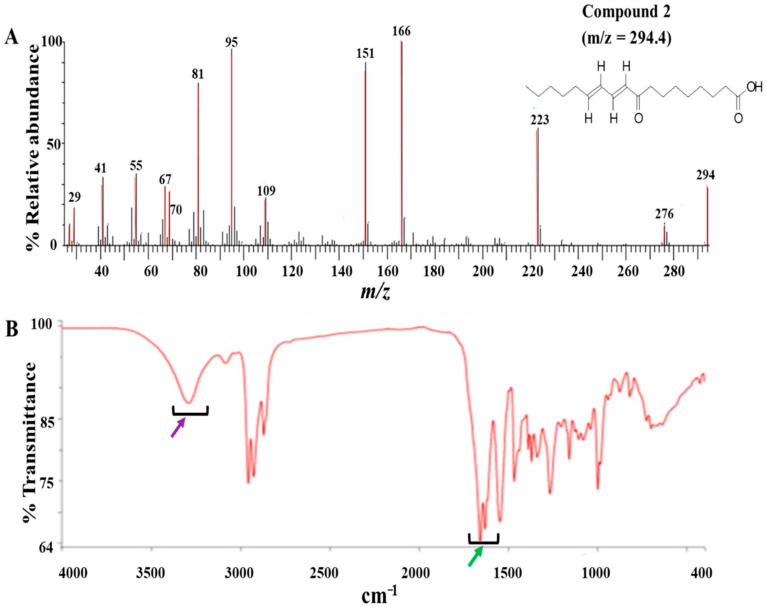
GC-MS and ATR-IR analysis of compound **2**: Compound **2** was isolated from the dichloromethane fraction of *Z. zanthoxyloides* (root) and identified through gas chromatography-mass spectrometric (GC-MS) analysis. The mass fragmentation of compound **2** identified it as 9-oxo-ODA with *m*/*z* = 294.40 (**A**). The IR spectrum displayed absorbance intensities within a range of wavenumbers suggestive of carboxyl (1760–1690 cm^−1^) (**B**, green arrow) and hydroxyl stretches (3500–3200 cm^−1^) ((**B**), purple arrow).

**Figure 2:**
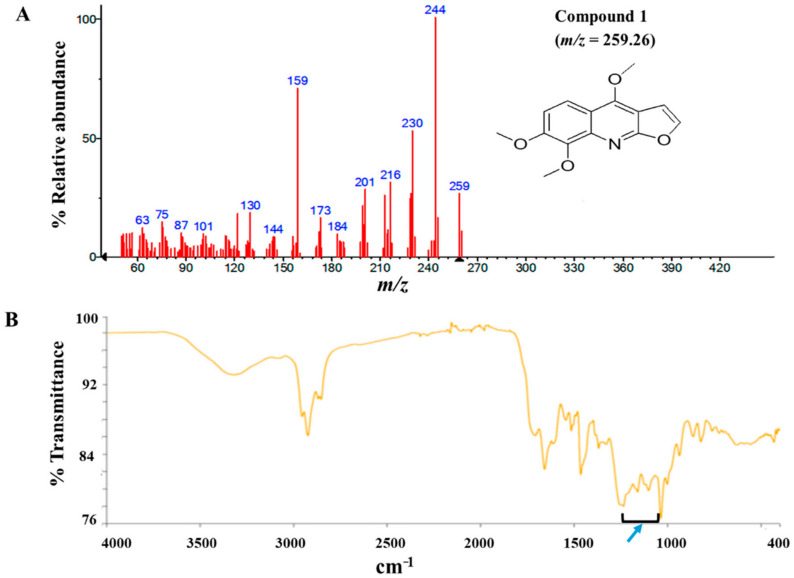
GC-MS and Attenuated Total Reflectance (ATR)-IR analysis of compound **1**: Compound **1** was isolated from the dichloromethane fraction of *Z. zanthoxyloides* (root) and identified through gas chromatography-mass spectrometric (GC-MS) analysis. The mass fragmentation of compound **1** identified it as 4,7,8-trimethoxyfuro (2,3-b) quinoline with *m*/*z* = 259.26 (**A**). The aromatic nature of compound **1** was suggested by the absorbance at 1335–1250 cm^−1^ ((**B**), blue arrow).

The modified Kupchan method of solvent extraction was employed to prepare solvent fractions of varying polarities from the crude extract of *Z. zanthoxyloides* (root) (Figure S2). Based on the promising antitrypanosomal activities of the dichloromethane fraction of *Z. zanthoxyloides* (root) reported in our previous study [14], the same solvent fraction was prioritized and subjected to gravity column chromatography to produce eleven column fractions that were tested for their antitrypanosomal activities in a 48-h cell viability analysis (Table 1). Based on their EC_50_ values, the most active column fractions were subjected to bioactivity-guided purification by liquid chromatography for subsequent analysis by GC-MS. This resulted in the isolation of compound **1** (4.6 mg) and **2** (3.8 mg) from column fractions 5 and 4, respectively (Table 1). Both compounds were eluted no later than approximately 33 min in the HPLC (Figure S1). GC-MS identification of compounds occurred at *m*/*z* of approximately 259 (4,7,8-trimethoxyfuro (2, 3-b) quinoline: compound **1**) and 294 (9-oxo-10, 12-octadecadienoic acid (9-oxo-ODA): compound **2**), respectively (Figure 1A and Figure 2A). Interpretation of mass spectra was conducted using a robust database of National Institute of Standard and Technology (NIST). Chromatographic elution of compounds in the GC-MS analysis occurred at retention times of 6.04 (compound **2**) and 34.14 min (compound **1**) (Figures S4 and S5). The IR spectra of the compounds also displayed absorbance intensities within a range of wavenumbers suggestive of carboxyl (1760–1690 cm^−1^) and hydroxyl stretches (3500–3200 cm^−1^) as evident in compound **2** (Figure 1B), while the presence of an aromatic amine functionality (1335–1250 cm^−1^) confirmed the aromaticity of compound **1** (Figure 2B).

Eleven gravity column chromatographic fractions of the dichloromethane fraction of *Z. zanthoxyloides* were each tested for their antitrypanosomal activities. Mean EC_50_ values and standard errors (SE) were calculated from three distinct experiments. Compounds **1** and **2** were subsequently isolated from fractions 5 and 4, respectively. DA = Diminazene aceturate (an antitrypanosomal drug); SEM = standard error of the mean.

### 3.2. Compounds ***1*** and ***2*** Were Selectively Antitrypanosomal

**Figure 3:**
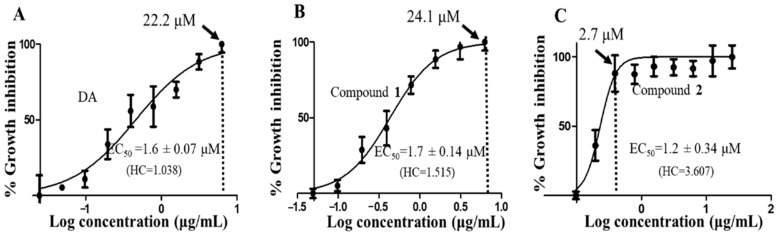
Dose-response curves of compounds. The dose-response curves and corresponding half-maximal effective concentration (EC_50_) values for DA (**A**), compound **1** (**B**) and compound **2** (**C**) were modeled from a non-linear regression analysis using the Hill function. Error bars were calculated from triplicate values. DA = diminazene aceturate; HC = hill coefficient; dotted vertical line = concentration of maximum growth inhibition of *T. brucei*.

To determine the antitrypanosomal potency and effects of the compounds on *T. brucei*, a 48-h alamarBlue cell viability assay was used. As depicted in their dose-response curves, both compounds exhibited significant antitrypanosomal potencies that were comparable to that of the animal African antitrypanosomal drug, diminazene aceturate (DA), which was used as the positive control (compound **1**, EC_50_ = 1.7 µM; compound **2**, EC_50_ = 1.2 µM; DA, EC_50_ = 1.6 µM). Compound **1** and **2** caused maximum inhibition of parasites at 24.1 µM and 2.7 µM at Hill coefficients of 1.515 and 3.607, respectively (Figure 3B,C). For compound **2**-treated parasites, inhibition of cells remained fairly constant beyond 2.7 µM, thereby corresponding to a relatively narrow window of inhibition at a higher Hill coefficient (Figure 3C). In the presence of normal mouse macrophages (RAW 264.7), compound **2** was more selective to *T. brucei* than compound **1** as evidenced by their selectivity indices (SI) (compound **1**, selectivity indices (SI) = 74.9; compound **2**, SI = 107.3; DA, SI = 86.7) (Table 2).

Selectivity profiles of both compounds against normal macrophages (RAW 264.7) compared to DA (compound **1**, SI = 74.9; compound **2**, SI = 107.3; DA, SI = 86.7), where SI (selectivity index) was calculated as the ratio of the EC_50_ value in RAW 264.7 cell lines to that in *T. brucei*. SEM = standard error of the mean.

### 3.3. Compounds ***1*** and ***2*** Induced Cell Cycle Arrest in T. brucei

**Figure 4:**
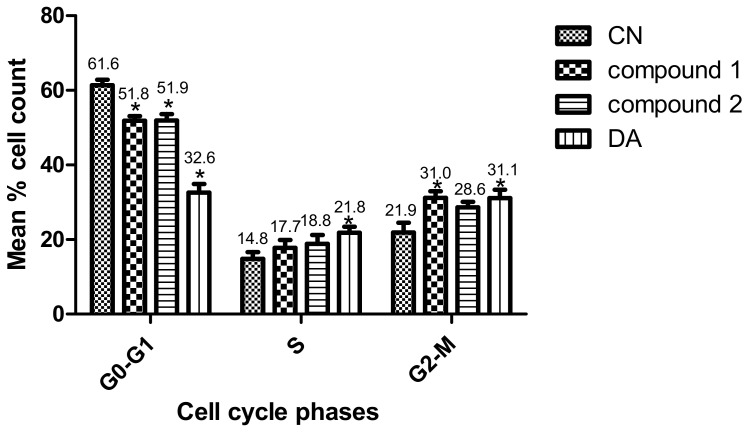
Effect of compounds on cell cycle of *T. brucei*. Error bars originate from mean percentage count ± standard error of the mean (Mean ± SEM) for three distinct experiments. Three cell cycle phases were analyzed: G0-G1, S and G2-M. Experiment was carried out at the EC_50_ values. DA = Diminazene aceturate; CN = Negative control (*: *p*-value < 0.05).

Parasites were challenged with compounds at the EC_50_ values to investigate their effects on the cell cycle. Three major cell cycle phases of *T. brucei* were identified: G0-G1, S and G2-M (Figure 4 and Figures S3). In comparison to untreated cells, there was a 9.6% (*p* = 0.007) and 9.5% (*p* = 0.010) reduction of G0-G1 cells in compound **1**- and compound **2**-treated cells, respectively (Figure 4). Despite the corresponding increase in G2-M cells, only the compound **1**-mediated effect was significant (compound **1**, 9.1% (*p* = 0.040); compound **2**, 6.7% (*p* = 0.080)). Moreover, the percentage increase in S phase cells when compared to the negative control was not significant for both compounds (compound **1**, 2.9% (*p* = 0.360); compound **2**, 4.0% (*p* = 0.260)). However, while the S phase was generally arrested by compound **2,** compound **1** blocked the progression of the G2-M phase. The population of S and G2-M phases were more distinctly resolved for compound **1** than compound **2**-treated cells (Supplementary Figure S3). Collectively, these results corresponded to a cell cycle arrest of S or G2-M phase that resulted in the reduction of *T. brucei* cells in G0-G1 (Figure 4 and Figures S3).

### 3.4. Compounds **1** and **2** Aggregated and Distorted Morphology and Distribution of T. brucei

**Figure 5:**
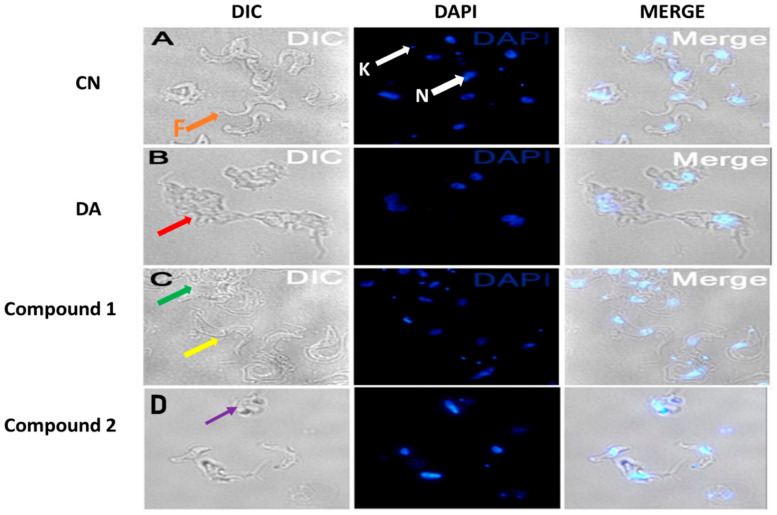
Effect of compounds on cell morphology of *T. brucei.* Cells were treated at the EC_50_ values of compounds. Red arrow (**B**) = aggregated DA-treated cells, green arrow (**C**) = aggregated compound **1**-treated population, yellow arrow (**C**) = less distorted compound **1**-treated population, purple arrow (**D**) = severely distorted compound **2**-treated population, F (orange arrow, (**A**)) = flagellum, K (white arrow, (**A**)) = kinetoplast, N (white arrow, (**A**) = nucleus; DIC = differential interference contrast; DAPI = 4′, 6-diamidino-2-phenylindole; DA = diminazene aceturate; CN = negative control.

Parasites challenged for 24 h at the EC_50_ values of compounds were observed for morphological changes by reflected fluorescence using DAPI. The elongated slender shape of *T. brucei* with helical flagella was observed under untreated conditions (Figure 5A). Compound **1** was relatively selective in effect as contrasted to the damaging nature of compound **2** (Figure 5C,D). This manifested as variations in the extent of damage to distinct populations of compound **1**-treated parasites (Figure 5C). Despite the severe aggregation of specific subpopulations in compound **1**-treated cells, other population of parasites were morphologically similar to untreated cells (Figure 5C). The flagella of most distorted parasites lacked the normal slender spiral shape typical of untreated cells.

### 3.5. Oxidant Capacity of Compounds in T. brucei

**Figure 6:**
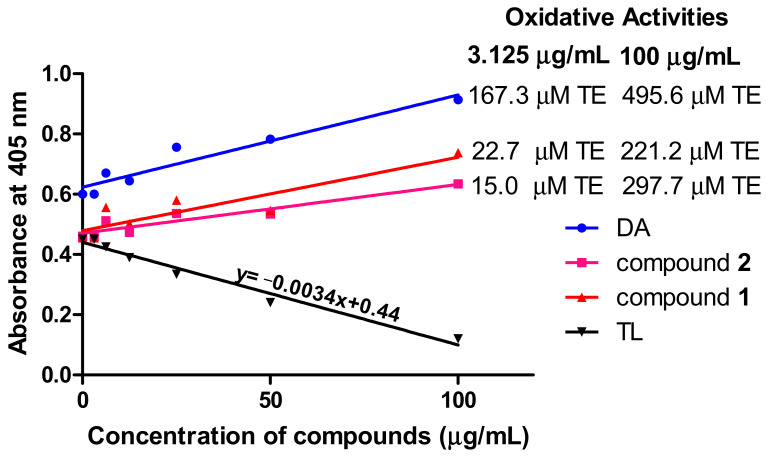
Oxidant capacity of compounds in *T. brucei*. In contrast to the standard antioxidant Trolox, the compounds were oxidative in their antitrypanosomal activities. Oxidative activities of compounds were calculated from the equation of the standard Trolox curve. DA = diminazene aceturate; TL = Trolox; TE = Trolox equivalent.

Measurement of reactive oxygen species is important in the investigation of oxidative damage in kinetoplastids. To investigate the oxidative potential of the compounds, their antioxidant properties in *T. brucei* were determined by employing the reducing properties of ABTS (2,2′-azino-bis (3-ethylbenzthiazoline-6-sulfonic acid). For an effective standard antioxidant, such as the water-soluble analog of vitamin E (Trolox), a dose-dependent reduction in absorbance of the ABTS radical at 405 nm is expected (Figure 6). Thus, the dose-dependent increase in absorbance for compound **1**- and compound **2**-treated cells is a strong indication of their oxidative rather than antioxidant potential (Figure 6). Compound **1** exhibited respective oxidative activities of 22.7 and 221.2 μM Trolox equivalent (TE) at the minimum (3.125 μg/mL) and maximum (100 μg/mL) tested concentrations of the compound, while compound **2** induced minimum and maximum oxidative activities of 15.0 and 297.7 μM TE, respectively, at the same tested concentrations (Figure 6). DA exhibited particularly high oxidative potentials of 167.3 and 495.6 μM TE at the minimum and maximum tested concentrations, respectively (Figure 6).

### 3.6. Effects of Compounds on Growth Kinetics of T. brucei

**Figure 7:**
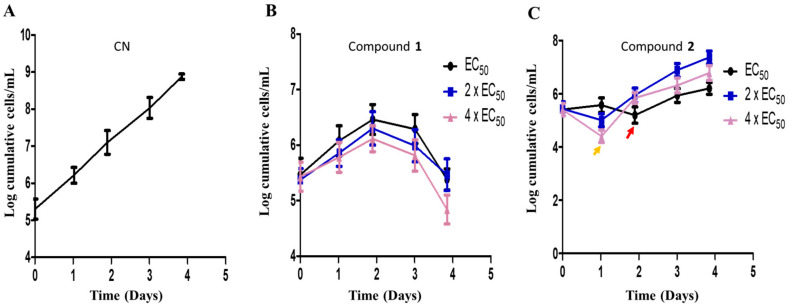
Compound sensitivity analysis in *T. brucei.* The growth kinetics of *T. brucei* was investigated in the presence of compounds **1** (**B**) and **2** (**C**) for 96 h at EC_50_, 2 × EC_50_ and 4 × EC_50_. Red arrow = growth profile at EC_50_ after 48 h (**C**); orange arrow = growth profile at 2 × EC_50_ and 4 × EC_50_ after 24 h (**C**); CN = negative control (**A**).

The metabolic kinetics of compounds **1** and **2** in the context of their sensitivities in *T. brucei* were investigated in order to assess their therapeutic potential towards drug discovery. The cumulative growth of parasites were monitored for 96 h at EC_50_, 2 × EC_50_ and 4 × EC_50_ values of compounds. In the absence of any antitrypanosomal, there was no effect on the cumulative growth curve of *T. brucei* (Figure 7A). In the presence of compound **1**, a gradual but consistent growth inhibition of parasites was observed over 96 h at different doses without any resumption of growth (Figure 7B). However, resumption of growth in compound **2**-treated cells was observed at different doses (Figure 7C). After the addition of compound **2** on the first day, immediate reduction in the cumulative number of cells at 24 h was highest for 4 × EC_50_ (Figure 7C). After this initial reduction in the number of cells, a loss of growth inhibition was observed after 24 h at 2 × EC_50_ or 4 × EC_50_ (Figure 7C). After this initial reduction in the number of cells, a loss of growth inhibition was observed after 24 h at 2 × EC_50_ or 4 × EC_50_ (Figure 7C). After 24 or 48 h, the addition of a new dose of compound **2** did not significantly reduce the number of parasites despite a general inhibition of the growth rate when compared to the negative control (Figure 7C). This was the general observation up to approximately 96 h (Figure 7C).

## 4. Discussion

The plant *Z. zanthoxyloides* is an important medicinal species in West Africa and the African continent at large. It is endowed with pharmacologically useful phytochemicals, some of which are responsible for their previously reported antitrypanosomal effects [14]. Even though compound **1** has previously been isolated from *Z. zanthoxyloides* [27,28], as well as shown to possess antitrypanosomal activity against *T. b. rhodesiense* and *T. cruzi* in a separate study [29], the complete structures and mechanisms of antitrypanosomal action for the phytochemicals of *Z. zanthoxyloides* have received little attention. In this study, two major phytochemicals, compound **1** (skimmianine) and compound **2** (9-oxo-10,12-octadecadienoic acid (9-oxo-ODA)) were isolated from the root of *Z. zanthoxyloides* and subsequently investigated for mechanisms by which they alter the cell viability, cell cycle, and cell morphology of *T. brucei.*

Compound **1** is a known inhibitor of acetylcholinesterase activity [16], while compound **2** was shown to activate the expression of PPARα target genes responsible for the β-oxidation of fatty acids [22]. Even though mammalian hosts infected with *T. brucei* may upregulate the expression of acetylcholinesterase [30,31], the expression of the enzyme has not been directly reported in *T. brucei*. The observation of the acetylcholinesterase’s activity in *T. evansi* provides the possibility that it may be encoded in *T. brucei* [32], but only a few hypothetically conserved orthologs of unknown functions have been identified in *T. brucei* (https://tritrypdb.org/). As key regulators of lipid metabolism in *T. cruzi* [23], PPARs may also play similar regulatory roles in *T. brucei* although no functional orthologs have been identified. Hence, aside the possible interferences of acetylcholinesterase and PPARα activities, other biochemical pathways of action for compound **1** and **2** cannot be overruled.

The present study suggests that oxidative damage contributes significantly to the mechanism of antitrypanosomal activities of compounds **1** and **2**. Oxidative stress involves a shift in the balance between oxidants and antioxidants in cells, with reactive oxygen species serving as key oxidants [33]. The mechanism of action of quinoline derivatives in *Leishmania* has been reported to involve mitochondrial oxidative stress [34]. The dihydroquinoline derivative OSU-40 (1-benzyl-1,2-dihydro-2,2,4-trimethylquinoline-6-ylacetate) was shown to exhibit its antitrypanosomal activity in *T. brucei* through the induction of oxidative stress [35]. Indeed, protection from oxidative stress by selenophosphate synthase is critical to the survival of *T. brucei* [36]. Moreover, the production of reactive oxygen species via lipid oxidation is known to be an important step in the biosynthesis and metabolism of oxylipins [19]. Hence, the observed oxidative activities of compounds **1** and **2** in this study may be a confirmation of a fundamental route by which this group of compounds exhibit their antiparasitic effects.

The utilization of oxidative stress as a means of action could be disadvantageous to host cells due to the potential toxicity associated with it. This may be demonstrated by nifurtimox and diminazene, which are commercially available drugs used in the treatment of human and animal African trypanosomiasis, respectively. The mode of action of nifurtimox is thought to involve the production of free radical and non-radical forms of reactive oxygen species and the subsequent induction of oxidative stress that may damage proteins, DNA, and lipids [37]. In the same way, by virtue of the high oxidative potential observed in this study, oxidative stress may contribute to the toxicity associated with diminazene as reported through damage to the kidney, liver, and brain [38].

Response to antimicrobial agents is generally associated with the development of tolerance, resistance, or persistence in pathogens [39]. While resistance is typically understood in terms of well-defined inhibitory concentrations of the antimicrobial, tolerance is better distinguished by a relatively longer duration for killing [39]. Furthermore, even though resistance to commercially available antitrypanosomal drugs is on the rise, the mechanisms are poorly understood despite the considerable progress [24,25]. In view of the fact that persistence is typically restricted to a limited subpopulation of the pathogen [39], a reduction in parasite sensitivity in the form of tolerance may be the more likely factor as far as the resumption of growth in compound **2**-treated cells is concerned. With a minimal amount of viability, tolerant parasites could easily bounce back despite the significant reduction in number or viability of parasites initially observed. In the context of tolerance, higher doses of 2 × EC_50_ and 4 × EC_50_ may have triggered a faster rate of parasite susceptibility to compound **2** and a subsequently effective recovery from growth inhibition as compared to the EC_50_ dose. It may be the case that high doses of compound **2** employed in this study met the minimum threshold required for the generation of tolerant *T. brucei* strains at a more efficient rate. However, further studies are required to confirm a permanent loss of sensitivity with regards to any inherited mutations and biochemical interactions between compound **2** and *T. brucei.*

A measure of consistency in growth inhibition at different concentrations of an antitrypanosomal agent may be a reflection of the compound’s metabolic stability. The metabolic conditions in *T. brucei* with regards to the presence or absence of unique protective enzymes and specific pH could be considerably different from that of the growth medium. A chemically intact compound could be inhibited or rendered inactive by specific metabolic enzymes as soon as it is transported into the parasite, thereby shortening its half-life or narrowing the window of inhibition in *T. brucei*. Thus, even though chemical or structural stability may contribute significantly to metabolic stability, the latter is not necessarily limited to or dependent on the former. The gradual but consistent inhibition of parasites in compound **1**-treated cells was in contrast with the relatively random resumption of growth in compound **2**-treated parasites at different concentrations, of which the metabolic stability of compound **2** within *T. brucei* may have partly contributed. Moreover, the structural instability of oxylipins, partly due to the susceptibility to lipid oxidation under enzymatic or non-enzymatic conditions, is well-documented [40], and this may further worsen the ability of compound **2** to remain active for a long period at physiological conditions in the parasite. It is by virtue of their chemical nature that new methods have been devised to improve the extraction and storage of oxylipins that would be helpful to consider in future studies of compound **2** [40]. However, successful identification of the potential intracellular factors would provide considerable insights into antitrypanosomal-parasite interactions required to understand the relative contributions of stability to the resumption of growth in compound-**2** treated cells. Future studies should therefore focus on the investigation of antitrypanosomal stability and sensitivity at varying conditions of temperature and pressure.

The arrest of the S and G2-M phase progression suggests that the compounds inhibited karyokinesis and cytokinesis, which led to a distortion and aggregation of *T. brucei*. Even though both compounds distorted the long slender morphology of *T. brucei*, the impact from compound **1** was relatively selective. This is consistent with the broad window of inhibition at low Hill coefficient, which has the potential of conferring varying degrees of effects on different subpopulations of parasites and might be a contributing factor. On the other hand, the narrow window of inhibition coupled with the high Hill coefficient observed for compound **2**-treated parasites would increase the damaging effects on parasites only within that window. Even though the parasites with the altered morphology appear to be severely damaged, some level of viability that is not detectable by fluorescence microscopy could still be present. This is so because in principle, an average of approximately 50% viability is lost in the overall parasite population at the half-maximal effective concentration (EC_50_), thereby suggesting that the viability of the parasites identified in the fluorescence microscopy may not necessarily represent the viability of the entire parasite population. Despite the morphological distortions, some populations of the parasite may still retain some level of viability at the EC_50_. Thus, an antitrypanosomal compound that fails to keep cells inhibited (either through a loss of metabolic stability or parasite sensitivity) may result in resumption of growth of cells after a particular point in time if a minimal amount of parasite viability is retained. That notwithstanding, it would still be beneficial to follow up with single trypanosomes in future studies to directly confirm the recovery of the normal slender shape under physiological conditions over relatively long periods.

## 5. Conclusions

This study demonstrates the chemotherapeutic properties of 9-oxo-ODA and skimmianine against African trypanosomes. It is the first time 9-oxo-ODA is isolated from the root of *Z. zanthoxyloides* and shown to possess selective antitrypanosomal activity. The results also indicate that oxidative damage may be an important mechanism by which 9-oxo-ODA and skimmianine inhibit the growth, alter the morphology, and arrest the cell cycle of *T. brucei.* The study paves the way for pharmacological evaluation of furoquinolines and oxylipins that may provide insights to facilitate the development of commercially available antitrypanosomal drugs.

## Figures and Tables

**Table 1:** Antitrypanosomal activities of chromatographic dichloromethane fractions of *Z. zanthoxyloides* (root).

Column Fractions	Mean EC_50_ ± SEM (μg/mL)
1	11.2 ± 0.71
2	13.4 ± 0.47
3	9.3 ± 1.73
4	2.0 ± 0.20
5	6.2 ± 1.30
6	9.7 ± 1.23
7	17.2 ± 3.37
8	8.9 ± 0.74
9	19.5 ± 2.12
10	28.3 ± 1.06
11	54.5 ± 1.12
DA	0.5 ± 0.05

**Table 2:** Antitrypanosomal activities and selectivities of compounds.

Compounds	Mean EC_50_ ± SEM (μM)		SI
	*T. brucei*	RAW 264.7	
Compound **1**	1.7 ± 0.14	127.3 ± 3.0	74.9
Compound **2**	1.2 ± 0.34	128.8 ± 2.3	107.3
DA	1.6 ± 0.07	138.7 ± 0.9	86.7

## Supplementary Materials

The following are available online at https://www.mdpi.com/2218-273X/10/12/1670/s1. Figure S1: HPLC chromatogram for compounds **1** and **2**; Figure S2: Schematic diagram for modified Kupchan method of solvent partition; Figure S3: Typical cell cycle histograms for compounds **1** and **2**; Figure S4: Total ion chromatogram for compound **1** in GC-MS; Figure S5: Total ion chromatogram for compound **2** in GC-MS.

## Abbreviations

AT	African trypanosomiasis
*Z. zanthoxyloides*	*Zanthoxylum zanthoxyloides* (Lam.) Zepern. and Timler
9-oxo-ODA	9-oxo-10, 12-octadecadienoic acid
PPARα	peroxisome proliferator-activated receptor alpha
