# Supplementary material for: Isolation and Antitrypanosomal Characterization of Furoquinoline and Oxylipin from Zanthoxylum zanthoxyloides
Source: Biomolecules. 2020 Dec 13;10(12):1670. doi: 10.3390/biom10121670 (PMC7763825; doi:10.3390/biom10121670)
Supplement: Supplementary file 1 [file biomolecules-10-01670-s001.pdf]

Figure S1: HPLC chromatograms for compounds 1 and 2

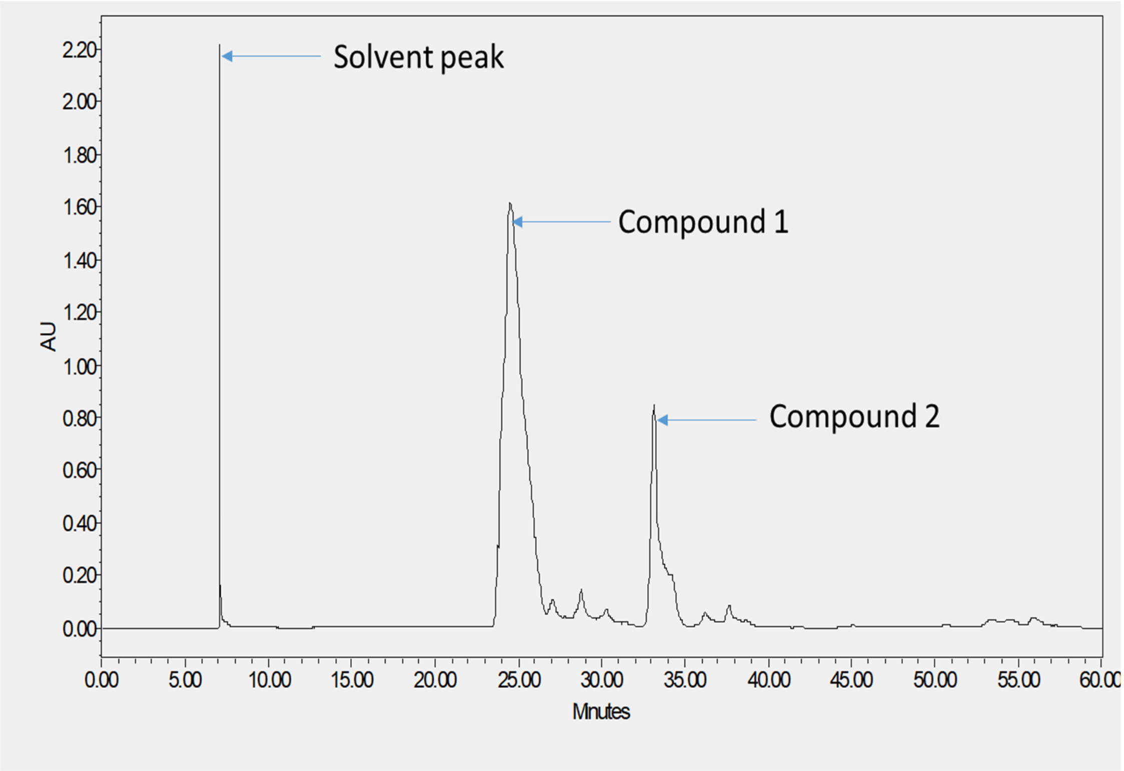

Both compounds were eluted not later than approximately 33 mins

Figure S2: Schematic diagram for modified Kupchan method of solvent partition

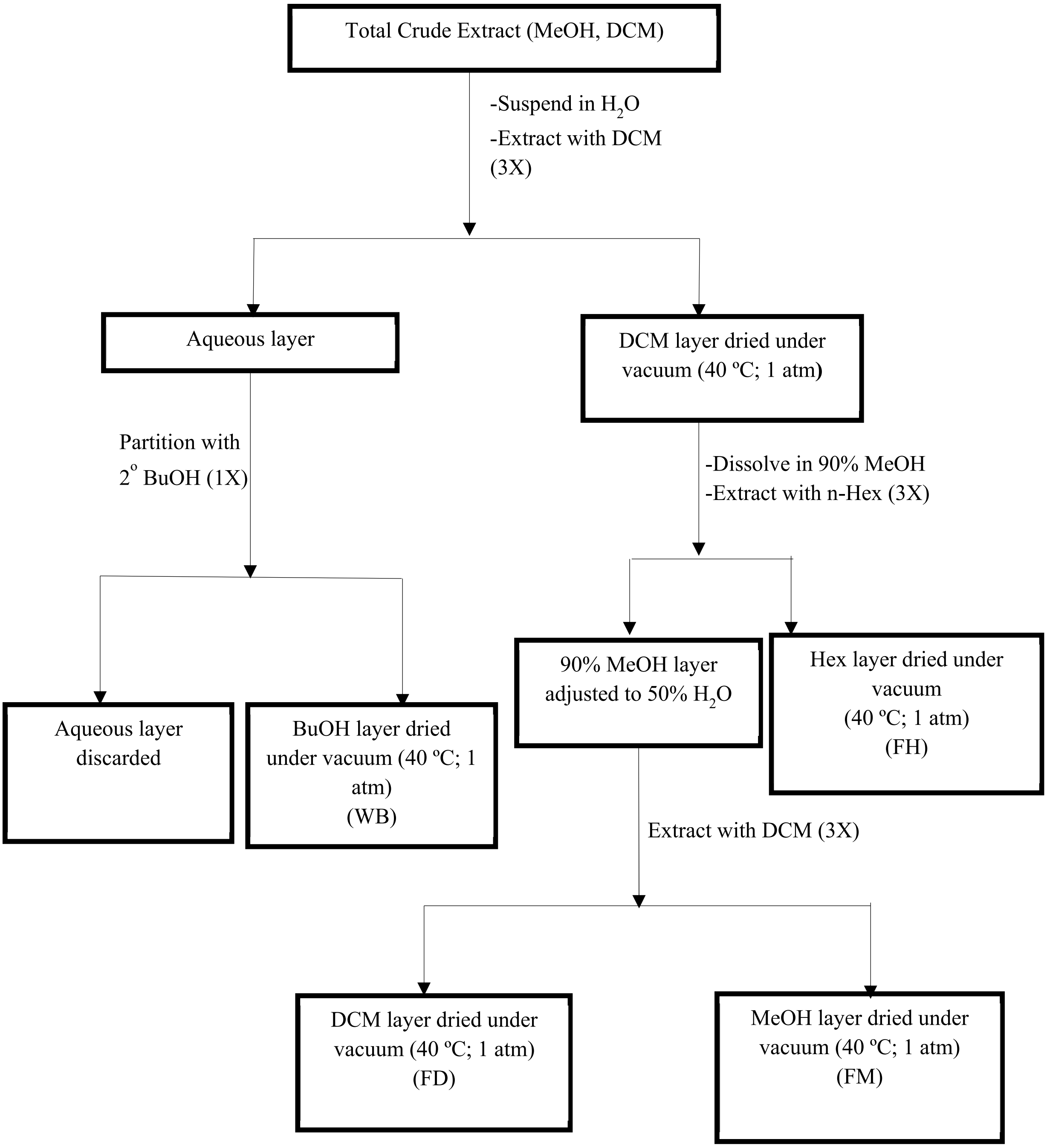

The pulverized air-dried plant material **(1 kg)** was soaked in absolute dichloromethane and left to percolate at room temperature for 1 week. The dichloromethane extracts were decanted and filtered through a mixture of cotton and glass wool. The plant material was then soaked in absolute methanol for another week after which the methanol extract was also decanted and filtered. Methanol and dichloromethane extracts were combined and dried under vacuum with a Heidolph Rotavap at 40 °C and 1 atm pressure to give a total crude extract **(TCE, 4 g)**. The TCE was suspended in water and extracted three times with dichloromethane. The remaining aqueous layer was then extracted once with sec-

butanol and the butanol fraction was dried under vacuum to give the water-butanol **(WB, 1.5 g)** fraction. The dichloromethane layer was dried under vacuum to give the extract which was suspended in a 9:1 mixture of methanol and water. This fraction was extracted three times with hexane after which the hexane layer was dried under vacuum to give a hexane fraction **(FH, 0.6 g)**. The remaining 9:1 mixture of methanol and water layer was phase adjusted to a 1:1 mixture and extracted three times with dichloromethane which was dried under vacuum to give a dichloromethane fraction **(FD, 0.3 g)**. The 1:1 methanol and water layer was also dried under vacuum to give a methanol fraction **(FM, 0.25 g)**. Four fractions were thus prepared from the root of *Z. zanthoxyloides*: water-butanol fraction (WB), hexane fraction (FH), dichloromethane fraction (FD) and methanol fraction (FM). MeOH=methanol; DCM=dichloromethane, BuOH=butanol; Hex=n-hexane.

Figure S3: Typical cell cycle histograms for compounds 1 and 2

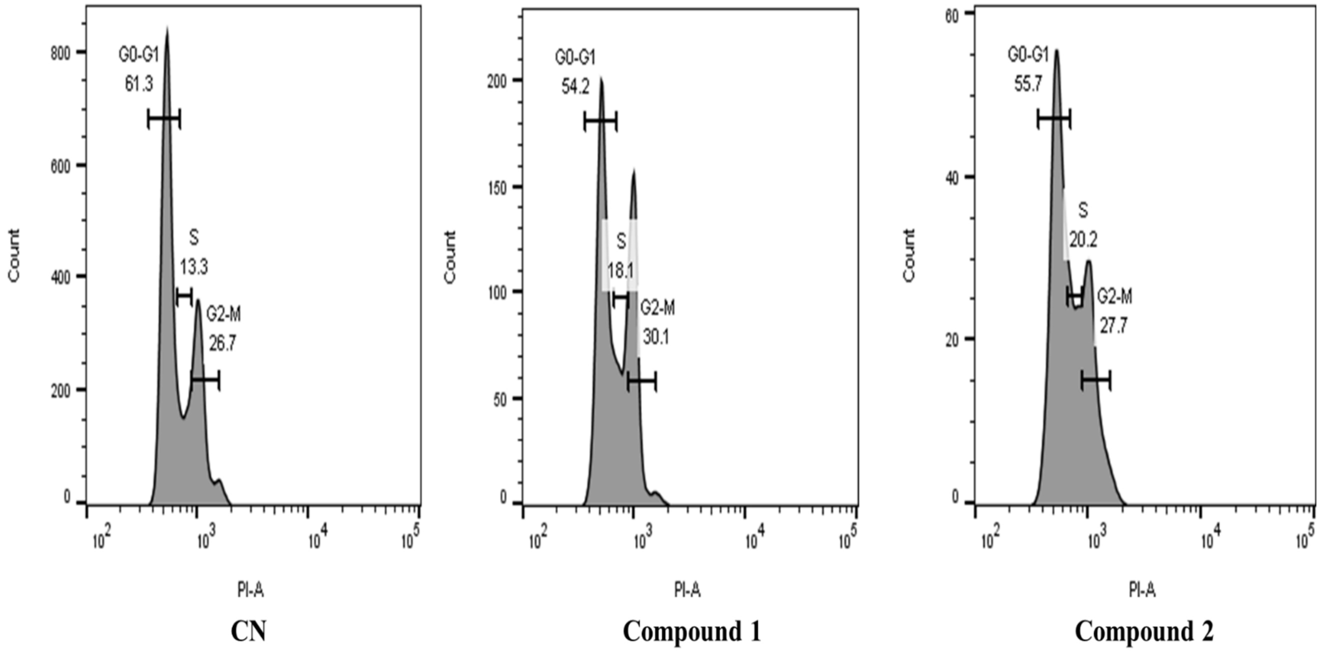

The histogram compares population count of parasites with intensity of propidium iodide. Three phases of the parasites were identified: G0-G1, S and G2-M. Experiment was carried out at the EC_{50} values. Both compounds induced a G0-G1 inhibition and a G2-M phase arrest of *T. brucei.* PI= propidium iodide; CN=Negative control.

Figure S4: Total ion chromatogram for compound 1 in GC-MS

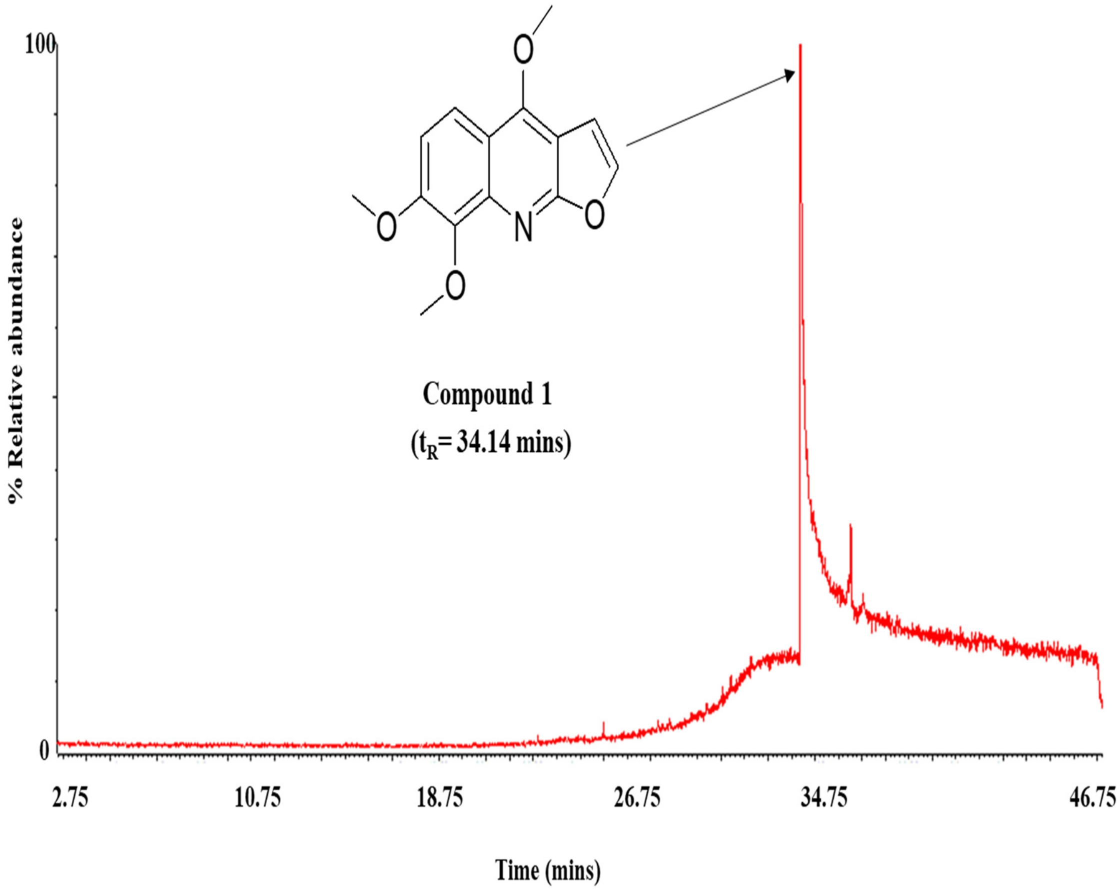

Compound 1 was eluted at a retention time of 34.14 mins

Figure S5: Total ion chromatogram for compound 2 in GC-MS

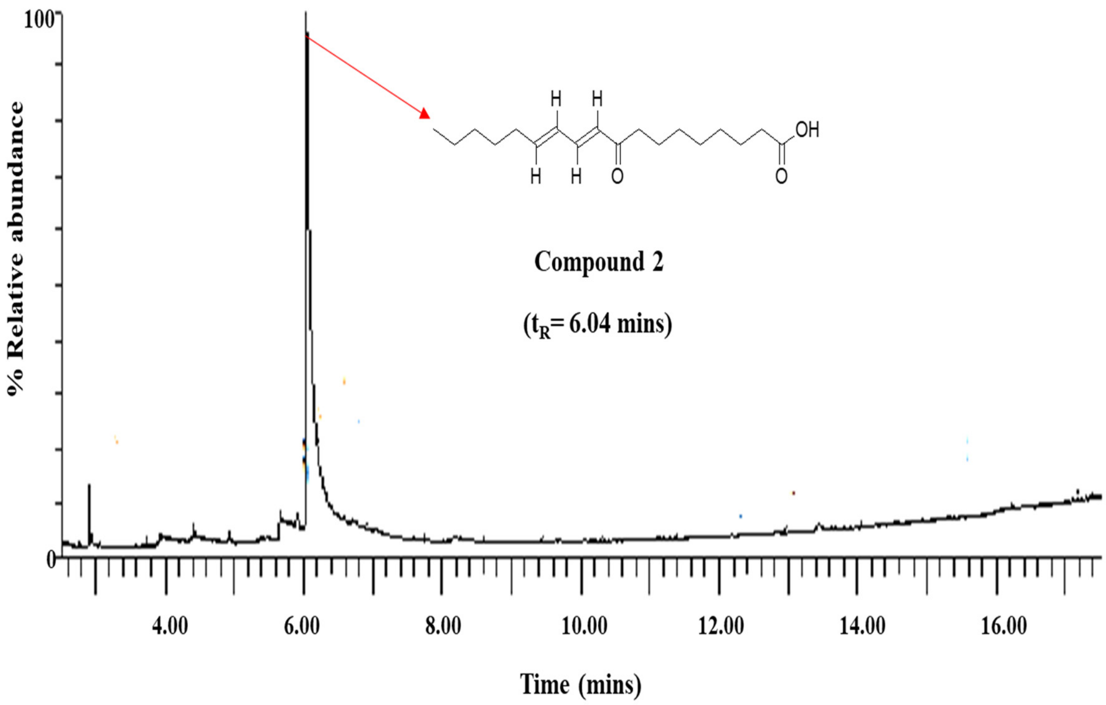

Compound 2 was identified at a retention time of 6.04 mins
